# Graft versus Host Disease in the Bone Marrow, Liver and Thymus Humanized Mouse Model

**DOI:** 10.1371/journal.pone.0044664

**Published:** 2012-09-05

**Authors:** Matthew B. Greenblatt, Vladimir Vbranac, Trevor Tivey, Kelly Tsang, Andrew M. Tager, Antonios O. Aliprantis

**Affiliations:** 1 Department of Immunology and Infectious Diseases, Harvard School of Public Health, Boston, Massachusetts, United States of America; 2 Center for Immunology and Inflammatory Diseases, Division of Rheumatology, Allergy and Immunology, Massachusetts General Hospital, Harvard Medical School, Charlestown, Massachusetts, United States of America; 3 Ragon Institute of Massachusetts General Hospital, Massachusetts Institutes of Technology, and Harvard and Division of AIDS, Harvard Medical School, Charlestown, Massachusetts, United States of America; 4 Department of Medicine, Division of Rheumatology, Allergy and Immunology, Brigham and Women's Hospital and Harvard Medical School, Boston, Massachusetts, United States of America; University of California, San Francisco, United States of America

## Abstract

Mice bearing a “humanized” immune system are valuable tools to experimentally manipulate human cells in vivo and facilitate disease models not normally possible in laboratory animals. Here we describe a form of GVHD that develops in NOD/SCID mice reconstituted with human fetal bone marrow, liver and thymus (NS BLT mice). The skin, lungs, gastrointestinal tract and parotid glands are affected with progressive inflammation and sclerosis. Although all mice showed involvement of at least one organ site, the incidence of overt clinical disease was approximately 35% by 22 weeks after reconstitution. The use of hosts lacking the IL2 common gamma chain (NOD/SCID/γc^−/−^) delayed the onset of disease, but ultimately did not affect incidence. Genetic analysis revealed that particular donor HLA class I alleles influenced the risk for the development of GVHD. At a cellular level, GVHD is associated with the infiltration of human CD4+ T cells into the skin and a shift towards Th1 cytokine production. GVHD also induced a mixed M1/M2 polarization phenotype in a dermal murine CD11b+, MHC class II+ macrophage population. The presence of xenogenic GVHD in BLT mice both presents a major obstacle in the use of humanized mice and an opportunity to conduct preclinical studies on GVHD in a humanized model.

## Introduction

Humanized mice have proven to be a valuable model that enables both the study of human-specific diseases such as HIV and the experimental manipulation of human cells *in vivo* [1], [2], [3]. In particular, the nonobese diabetic/severe combined immunodeficiency (NOD/SCID, hereafter NS) model, whereby immunodeficient NS or NOD *Rag2* ^−/−^ mouse hosts are reconstituted with human CD34+ cells, has been widely used and informative [2]. Advances on this model include the use of NOD/SCID/γc^−/−^ (hereafter, NSG) hosts where deficiency for the IL2 receptor gamma chain promotes improved engraftment [1], [4], [5]. Furthermore, surgical transplantation of human fetal liver and thymus alongside the engraftment of human bone marrow (hereafter BLT for bone marrow, liver and thymus) facilitates robust T cell and humoral immune responses by allowing T cells to be educated on syngenic human thymic tissue [1], [6], [7].

Here we report a major obstacle in the use of humanized mice. NS BLT and NSG BLT mice develop a spontaneous xenogenic graft versus host disease (GVHD) characterized by lymphocytic infiltration and sclerosis of the skin, GI tract, parotids and lungs, culminating in death. All NS BLT and NSG BLT mice examined displayed disease in at least one organ system. In considering humanized mouse models, GVHD both reduces the number of usable mice in a cohort and additionally complicates the results of disease models conducted in humanized mice by overlaying a second set of pathologies. Thus, as the technology of humanized mice as a platform to study T and B cells advances and donor cellular function improves, these advances will be met with commensurate increases in the incidence of GVHD. Along with these challenges, the occurrence of GVHD simultaneously presents an opportunity for the experimental study of GVHD and similar fibrotic autoimmune disorders, such as slceroderma, in a humanized system.

## Materials and Methods

### Generation and Housing of the BLT model

BLT mice were prepared as previously described [1]. Briefly, NOD/SCID or NOD/SCID/γ_c_^−/−^ mice 6–8 weeks old were sublethally irradiated with 2Gy, anesthetized, and 1mm fragments of human fetal thymus and liver (Advanced Bioscience Resources) were implanted under the kidney capsule, either unilaterally or bilaterally. Additionally, CD34+ cells were isolated from fetal liver via anti-CD34 microbeads (Miltenyi), and 1×10^5^ cells were injected intravenously within 6 hours after surgery. All surgery was performed under anesthesia and all efforts were made to alleviate any suffering associated with these procedures. All mice were subsequently housed in microisolator cages in a specific pathogen-free facility in accordance with Institutional Animal Care and Research Committee-approved protocols at Massachusetts General Hospital. The Massachusetts General Hospital Institutional Animal Care and Research Committee specially approved this study.

### Isolation of Skin Infiltrating CD4+ T cells and macrophages, and splenic T-cells

This was performed as previously described [8]. Approximately 10 cm^2^ pieces of back skin were shaved, washed with PBS, and digested for 1 hour with 0.27% collagenase type XI (Sigma) and 1 mg/10mL of hyularonidase type IV (Sigma) in RPMI medium. The resulting cell suspension was filtered over a 70 µm strainer, and stained with the listed antibodies for FACS on a FACS Aria (BD Biosciences, Franklin Lakes, NJ). All antibodies were from BD biosciences except for anti-human CD3 (Biolegend, San Diego, CA). RNA was isolated from sorted macrophages using qiazol (Quiagen). T cells were restimulated with PMA/ionomycin.

Alternatively, splenic CD4+ T cells were isolated using magnetic bead purification with anti-human CD4 (Miltenyi, Germany). Isolated CD4+ T cells were resimulated with PMA/ionomycin.

### Explant Cultures, ELISA, and autoantibody screen

Back skin from shaved GVHD and control mice was trimmed to 1 cm^2^ sizes and cultured overnight in RPMI-1640 with 10%FCS and antibiotics. For ELISA analysis, supernatants from either skin explants or T cell cultures were analyzed using Legend-Max kits from Biolegend for IL10, IFNγ, or IL4 or a kit from eBioscience for IL17. An autoantibody screen was conducted by the Brigham and Women's Hospital clinical laboratories using indirect immunofluorescence to evaluate the serum reactivity to the HEP2 cell line [9]. The HEP2 cell line was maintained internally by the Brigham and Women's Hospital clinical laboratories. Additionally, a clinical EIA was run to test for anti-Scl70 antibodies.

### Quantitative realtime PCR

RNA was isolated from the indicated cells or tissues by qiazol (Qiagen, Germany) and cDNA synthesized using a kit from Applied Biosciences. Realtime PCR was performed using the Brilliant II SYBR Green Master Mix (Stratagene, Santa Clara, CA) on a Mx3005P qPCR system (Stratagene).

## Results

### Gross and Histologic Characterization of GVHD in humanized BLT mice

**Figure 1:**
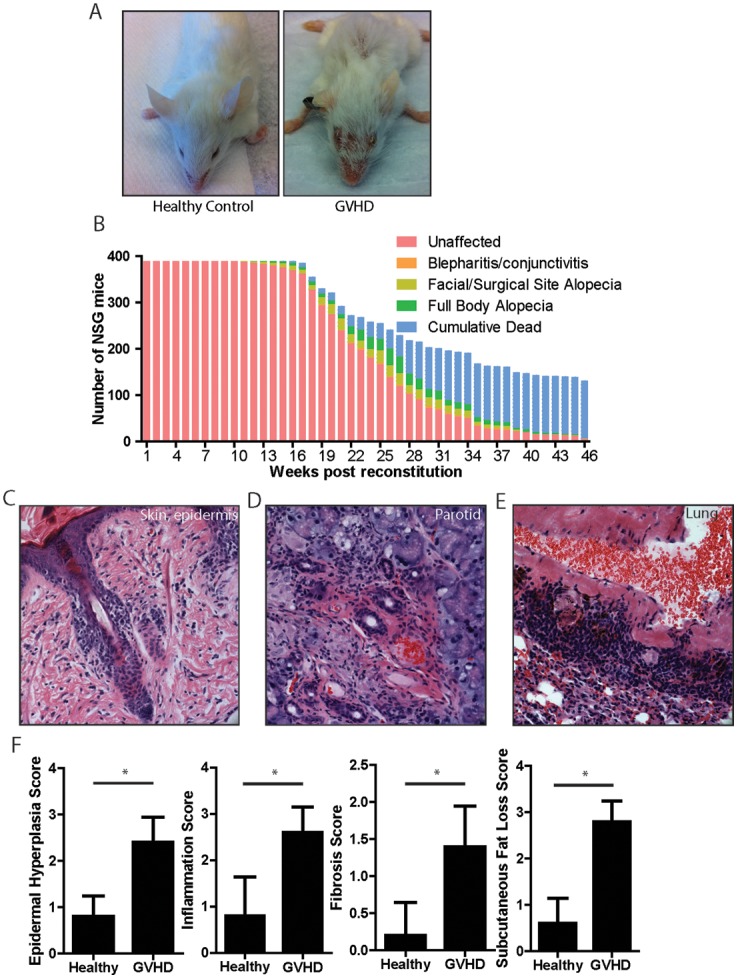
Clinical and Pathologic Description of GVHD in BLT mice. (A) Gross findings in BLT GVHD, demonstrating facial and trunk alopecia and blepharitis. (B) Kinetics of disease progression in NOD SCID γc^−/−^ mice. Initial n = 389. Subjects enrolled in specific experimental protocols were censored at the time of enrollment. (C–E) Representative histologic images of the indicated organs. 6 mice with GVHD and 6 healthy controls were analyzed. Original magnification, 100X. (F) Pathologic scoring of the skin, demonstrating that epidermal hyperplasia, inflammation, fibrosis and subcutaneous fat loss all correlate with the clinical manifestations of GVHD. “*” indicates p<.02 by a two-tailed Mann Whitney test. n = 5 mice per group.

GVHD in NOD SCID (NS) and NOD SCID γ_c_^−/−^ (NSG) BLT mice developed via a stereotypic order of events (Figure 1). First mice manifest blepharitis or conjunctivitis along with ruffled fur. Second, alopecia develops on either the face or at the site of the surgical scar from the implantation procedure (Figure 1A) and subsequently spreads to involve the entire body. Lastly, many affected mice succumb to the disease. Blepharitis develops starting at 12 weeks post reconstitution and mice succumb to disease starting at 15 weeks after reconstitution (Figure 1B). Histologically, skin involvement is characterized by a lymphocytic infiltrate of the epidermis, hair follicle, and dermal/subcutaneous junction with corresponding dropout of hair follicles, loss of subcutenaous fat, epidermal hyperplasia and hyalinization of dermal collagen, features similar to those observed in human GVHD or scleroderma (Figure 1C, Information S1). Occasional ulceration of the epidermis was also observed (Information S1). Blinded scoring of skin histology from BLT GVHD and donor-matched healthy control mice confirmed substantial increases in inflammation, epidermal hyperplasia, fibrosis and subcutaneous lipoatrophy scores in clinically affected animals (Figure 1D).

Just as GVHD is a systemic process in humans, several organs other than the skin are affected in BLT mice. The parotid glands were involved with a lymphocytic infiltrate and sclerosis surrounding central ducts (Figure 1D, Information S1). Colitis characterized by a lamina propria infiltrate was also present (Information S1). Notably, the lungs of all mice, even those without any overt clinical disease, all had a dense lymphocytic and macrophage-rich infiltrate sheathing the bronchi and pulmonary arteries, resulting in destruction and engulfment of the smooth muscle in the adventitia (Figure 1E, Information S1). No significant thryoiditis, pancreatitis or esophagitis was detected.

To evaluate for a humoral component of disease, the serum of NS BLT mice with GVHD was screened by indirect immunofluorescence for anti-nuclear antibodies (data not shown). No reactivity to Hep2 cells was detected. Anti-Scl70 antibodies are found in patients in a subset of patients with diffuse scleroderma as well as a fully murine model of chronic GVHD [10]. ELISA screening for serum anti-scl70 antibodies was negative in NS GVHD mice.

### The Contribution of Donor HLA class I alleles to the Development of GVHD

**Figure 2:**
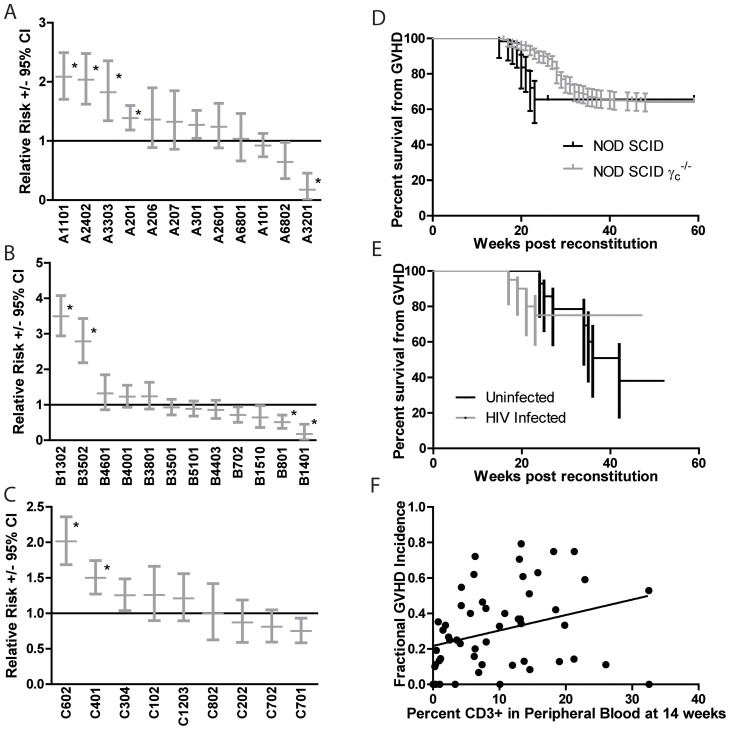
The influence of specific graft HLA class I alleles, host γ_c_ deficiency, HIV infection, and degree of reconstitution on the development of GVHD. (A–C) HLA class I data and the incidence of GVHD was recorded for each of over 1800 mice comprising 46 cohorts. Only those HLA class I alleles represented in at least 3 independent cohorts were included in the analysis. The relative risk for each HLA allele is displayed. Error bars represent the 95% confidence interval. Significance was determined by the chi-squared test with the Bonferroni correction applied for multiple comparisons. (D) The kinetics of GVHD onset were determined in NOD SCID and NOD SCID γc^−/−^ hosts. P<.05 by a Gehan-Wilcoxon test. For NOS SCID n = 61, for the NOD SCID γc^−/−^ group n = 343. Error bars represent the 95% confidence interval. (E) The effect of HIV infection on GVHD incidence in NSG hosts. p = ns by a Gehan-Wilcoxon test. n uninfected = 34, n HIV infected = 40. Error bars represent the 95% confidence interval. (F) The total percent of human CD3+ T cells present in the peripheral blood of humanized BLT mice at 14 weeks after reconstitution was plotted. Each dot represents the average percentage of CD3+ cells and the rate of GVHD present in a given cohort of mice reconstituted with a single donor. The line of best fit is plotted. The slope is. 008712 (95% confidence interval. 001348 to. 01608, P value .0212).

Specific donor HLA class I alleles have been identified as strong genetic modifiers of the incidence and manifestations of human GVHD [11], [12]. To determine the effects of HLA class I, 50 donor cohorts comprising approximately 1700 mice were typed for donor HLA class I alleles and data regarding the incidence of GVHD was collected (Figure 2A-C, Information S1). Contingency table analysis was used to calculate the relative risk of each specific HLA allele present in at least 3 independent cohorts. After chi-squared analysis and correction for multiple comparisons the following HLA Class I alleles showed a significant increase in the relative risk of GVHD: A1101, A2402, A3303, A201, B1302, B3502, C602, and C401. HLA A3201, B801, and B1401 showed a significant protective effect.

### The Effects of γ_c_ , HIV infection and lymphoid reconstitution on GVHD

In an attempt to identify hosts that would avoid the development of GVHD during the production of humanized mouse cohorts, the kinetics of GVHD was compared in NOD SCID (NS) and NOD SCID γ_c_^−/−^ (NSG) hosts. γ_c_ is a shared component of the receptor for IL-2, IL-4, IL-9, IL-15, and IL-21, and NSG mice have been used as humanized mouse hosts as they display improved engraftment of human hematopoietic stem cells [13], [14], [15], [16]. NSG hosts displayed a significant delay in the onset of GVHD of approximately 6 weeks. However the cumulative incidence of disease by 35 weeks after reconstitution was the same in both groups at about 35% (Figure 2D). Comparisons of disease in NS and NSG mice at the histologic and gene expression level did not reveal any significant differences (data not shown).

As a major use for humanized mice is to serve as an experimental platform for studies of HIV infection, the effects of HIV on GVHD incidence were studied (Figure 2E). HIV infection both promoted an approximately 8 week earlier disease onset and also reduced the total cumulative incidence in half versus uninfected, donor-matched controls. However, at the cohort size available (n = 34 uninfected, n = 40 HIV infected) these effects failed to reach statistical significance (P = .107 by a Mantel-Cox test), perhaps due to the crossing hazard functions undercutting the power of analysis. This trend is consistent with a model whereby early HIV infection has an innate immune costimulatory function that promotes disease, and later HIV-mediated CD4+ depletion ultimately lowers the cumulative incidence.

The correlation between the degree of lymphoid reconstitution as measured as percentage CD3+ cells present in a peripheral blood sample 14 weeks after reconstitution and the rate of GVHD was compared between different donor cohorts (Figure 2F). As expected, there was a significant correlation between the degree of reconstitution and GVHD incidence, however it is notable that even cohorts with low to undetectable levels of lymphoid reconstitution developed GVHD at a detectable rate. This suggests that, as opposed to general T cell reconstitution, the presence of certain pathogenic T cell clonotypes that may be present below the limit of detection in peripheral blood and expand at the sites of pathology is the critical factor in the development of GVHD.

### Molecular Characterization of GVHD

**Figure 3:**
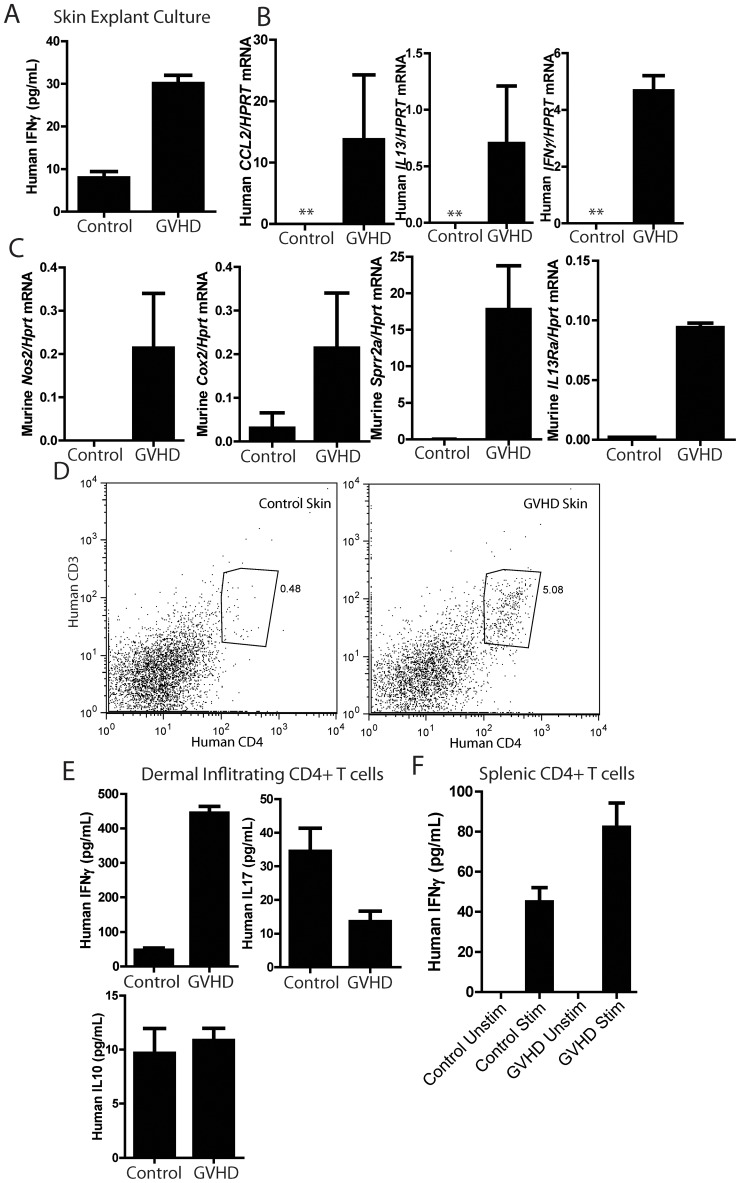
Characterization of T cells in BLT GVHD mice. (A) IFNγ ELISA on supernatants from skin explants cultured for 12 hours *ex vivo*. p = .0001 by a two-tailed Student's t-test. (B) Realtime PCR analysis for the indicated genes on RNA samples isolated from the skin of control and BLT GVHD mice. “**” indicates that the signal for both HPRT and the gene of interest were below the limit of detection. (C) Realtime PCR analysis for the indicated genes on RNA samples isolated from the skin of control and BLT GVHD mice. For all genes displayed, p<.05 by a two-tailed Student's t-test. (C) Flow cytometric analysis of cells isolated from the skin of BLT GVHD and control mice and stained for the indicated markers. (D) ELISA for the indicated cytokines on tissue culture supernatants of CD4+ T cells sorted from control and BLT GVHD skin as in (C) and restimulated with PMA and ionomycin. For IFNγ, P<.0001, for IL17, P = .0027, both by a two-tailed Student's t-test. (E) IFNγ ELISA on the tissue culture supernatants of CD4+ T cells isolated from the spleen of control and BLT GVHD mice by magnetic bead sorting and restimulated with PMA + ionomycin. Comparing stimulated healthy and GVHD T cells, P = .0016 by a two-tailed Student's t-test.

To explore the mechanisms of disease at the molecular and cellular levels, the cytokines produced by skin explants from affected and donor-matched controls were measured. Development of GVHD correlated with an approximately 3-fold increase in human IFNγ production (Figure 3A). Human IL4, IL17, IL21, IL23 and IL10 were all below the limit of detection in the same cultures (data not shown). Additionally, RNA was prepared from the skin of affected mice and controls (Figure 3B). One strength of the BLT GVHD system is that, by choosing human or murine specific primers, the expression of genes in the human immune infiltrate can be distinguished from that in the murine stroma or innate immune cells. A signal for a human housekeeping gene HPRT was absent in healthy BLT mice, demonstrating that human immune infiltrates are largely absent from the skin of healthy mice. This also precludes a relative comparison of expression levels between human genes in GVHD and healthy control mice. GVHD mice displayed robust expression of human IFNγ, and the pro-fibrotic mediators human IL13 and human CCL2 (Figure 3B) [8]. No murine IFNγ could be detected, arguing that human infiltrating immune cells are the sole source of IFNγ in the skin (data not shown). Development of GVHD induced increases in the expression of the inflammatory markers, murine *Cox2* and *Nos2* (Figure 3C). We have previously characterized that expression of several IL13 target genes correlates with the induction and severity of fibrotic skin diseases such as scleroderma [8]. Consistent with the fibrotic skin disease seen in the BLT GVHD mice and the above expression of IL13, expression of the IL13 target gene, murine *Sprr2a*, was increased (Figure 3C) [17]. Additionally, we have previously observed that expression of *IL13Ra1* correlated with an IL13 response signature in both a mouse model of sclerodermatous GVHD and in human scleroderma [8]. Consistent with the increase in *Sprr2a* levels, expression of murine *IL13Ra1* was also increased.

### Characterization of T cell and macrophage populations in GVHD

To directly examine the composition of the inflammatory infiltrate in the skin, skin was processed by collagenase digestion, and the resulting cell suspension was analyzed by FACS. Human CD4+ T cells were present in mice with clinical GVHD, but only small numbers were present in unaffected HLA-matched controls (Figure 3D). Human CD19+ B cells, CD8+ T cells, CD11c positive dendritic cells were not present at significant levels (data not shown). Skin infiltrating CD4+ T cells were sorted and restimulated in vitro and their cytokine secretion profile analyzed by ELISA (Figure 3E). Development of GVHD was associated with a substantial shift towards IFNγ production and away from IL17 production. IL4 secretion was below the limit of detection. A similar skewing towards increased IFNγ production was seen in CD4+ cells isolated from the spleen (Figure 3F). IL10 was detectable in both the restimulated skin and spleen cultures, but was unchanged in GVHD mice (Figure 3E and Information S1). IL21 was not produced at detectable levels in the same set of supernatants (data not shown). Taken together, this analysis supports a model whereby skewing CD4+ T cells towards Th1 differentiation promotes BLT GVHD.

**Figure 4:**
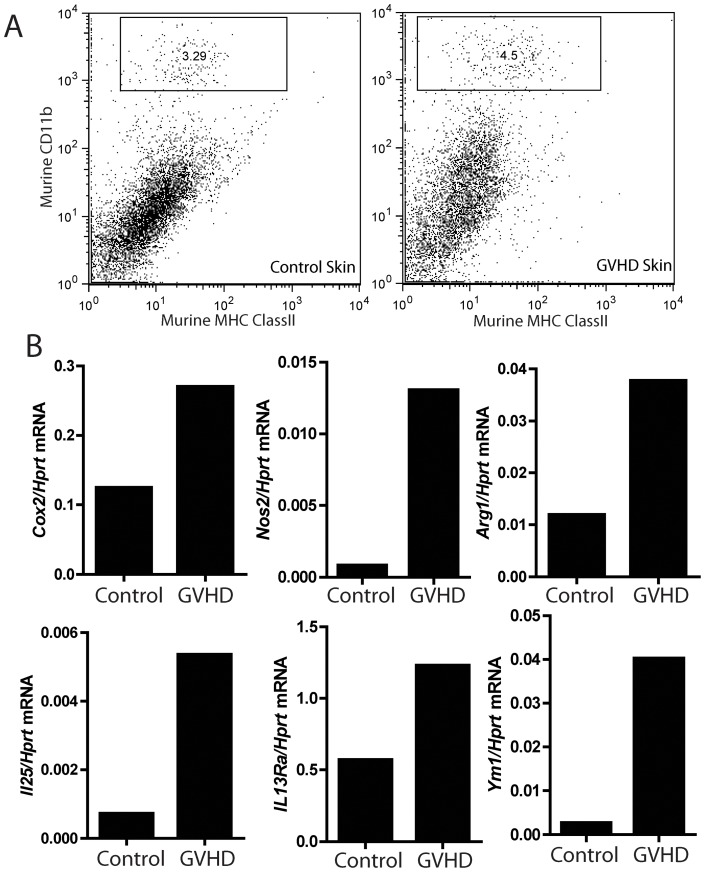
Characterization of a murine macrophage population in the skin of BLT GVHD mice. (A) Representative FACS plots showing the presence of a murine dermal macrophage population defined as CD11b^+^, MHC class II^+^. (B) Realtime PCR analysis for the expression of the indicated transcripts in macrophages sorted as in (A) from control and BLT GVHD mice.

Previously in a murine model of sclerodermatous GVHD, we identified a dermal CD11b+, MHC class II+ macrophage population that collaborates with infiltrating CD4+ T cells to produce pathology similar to that observed in the BLT mice with GVHD. In sclerodermatous GVHD this population of macrophages expressed a mixture of markers associated with M1 or “classical” (Cox2, Nos2) and M2 or “alternative” (Ym1) macrophage polarization [18]. A similar dermal CD11b+, MHC class II+ macrophage population was identified in the skin of BLT GVHD mice (Figure 4A), which also displayed a mixed M1/M2 macrophage polarization phenotype with increased expression of *Cox2, Nos2, Arg1* and *Ym1* (Figure 4B and Information S1). IL-25 has been suggested to function as a growth factor for a macrophage-like cell population that secretes pro-fibrogenic cytokines such as IL-13 [19], [20]. Macrophages in the skin of BLT GVHD mice displayed an upregualtion of IL25 (Figure 4B). Additionally, these cells also express *IL13Ra1* (Figure 4B), which is upregulated in GVHD, suggesting both that they are competent to respond to IL13, and as mentioned above, increased *IL13Ra1* was associated with an IL13-driven expression signature in murine GVHD and human scleroderma [8]. Despite the BLT model demonstrating robust reconstitution of human myeloid cells in the peripheral blood, secondary lymphoid organs, vaginal mucosa and GI tract, this macrophage population identified in the skin is entirely mouse in origin, based on the species specificity of the FACS and realtime PCR reagents used [21], [22].

## Discussion

Mice bearing a humanized immune system offer the flexibility to experimentally manipulate human cells in vivo and a non-primate host to model infectious agents such as HIV. Advances in the development of humanized mice, such as co-transplantation of human thymic and fetal liver tissue, have resulted in improved T cell engraftment and function [1], [6], [7]. However, here we report that a significant proportion of humanized mice develop clinical GVHD, with all animals demonstrating some degree of histopathologic disease. This untoward effect of reconstitution of immunodeficient mice with a human immune system is a major hurdle in the development of these models. Notably, other investigators have independently observed GVHD in humanized mice, indicating that this is a generalized phenomenon not likely to be attributable to the specific conditions used in this study [23]. With anticipated advances in reconstituting human T cell function in mice, we predict this issue will become progressively more severe. The insight we provide here into the potential pathogenic mechanisms at work will provide the basis for rational modification of these systems to reduce this limiting complication.

Previously we observed in a fully murine model of GVHD that the induction of a mixed M1/M2 phenotype in dermal macrophages is associated with fibrotic skin disease [8]. A similar phenotype has been observed with tumor-associated macrophages [24]. Interestingly, this observation is recapitulated in the BLT GVHD model, despite these mice displaying a significantly more pronounced Th1 polarization than observed in the purely murine sclerodermatous GVHD model. This suggests that factors outside of T-cell derived Th1/Th2/Th17-associated cytokines drive the acquisition of this phenotype. While traditional models of fibroinflammatory disease proposed that Th2 polarization promotes, and Th1 polarization inhibits, fibroinflammatory diseases, the Th1 predominance of this model support the more textured emerging view that, in collaboration with other factors, IFNγ can contribute to the development of fibroinflammatory diseases [25], [26], [27], [28], [29], [30]. Given that IFNγ appears to have a direct anti-fibrotic effect in several different systems, perhaps its role is more related to mediating key inflammatory and loss of tolerance steps in the process of disease development as opposed to being a direct driver of fibrosis [31], [32]. This is supported by the observation that human IFNγ does not cross react with murine cells. Thus, if IFNγ plays a functional role, it must be limited to its actions on human graft-derived cells [33]. Alternatively, it is possible that a Th1-derived factor other than IFNγ is the key cytokine in promoting GVHD in BLT mice. The difficulty in associating fibroinflammatory diseases purely with Th1 or Th2 differentiation may also be explained by the observation that the most potent Th2-associated pro-fibrotic cytokine, IL13, is additionally produced by innate immune cells and production by these sources may not be tied to Th1/Th2 skewing [24], [34]. Supporting this is our observation that expression of IL13 target gene *Sprr2a* is increased despite undetectable levels Th2 differentiation in T cells from BLT GVHD mice [8].

The strong association between the presence of human CD4+ T cells in the skin and GVHD suggests an obvious and direct model for their involvement in disease. Conversely, the association of donor HLA class I alleles with risk for GVHD suggests that CD8+ T cells also contribute. However, the absence of significant numbers of CD8+ T cells in lesional skin makes their mode of action less clear. One possibility is that CD8+ T cells are only present in the skin during early subclinical disease. A second possibility is that CD8+ T cells are involved in loss of tolerance within secondary lymphoid organs, functioning as an early source of IFNγ that promotes Th1 skewing of CD4+ T cells. The effects of donor HLA class I may also reflect the contribution of a class I-dependent NK cell mechanism [35], [36]. While many studies of the influence of specific donor HLA class I alleles were conducted on populations with little overlap with the donors in the present study, donor HLA C401 has been identified as promoting severe GVHD in both humans and the BLT GVHD model, suggesting that the BLT GVHD model may be predictive of specific genetic or environmental factors that contribute to the development of human GVHD [11]. From a practical perspective, knowledge of the relative risk conferred by various donor HLA class I alleles may facilitate pre-screening of donor tissue for those that induce a reduced burden of GVHD.

At the same time that GVHD poses a challenge for the use of humanized mice, it presents an opportunity to study GVHD or autoimmune-like pathology using human cells in vivo. In this respect, BLT GVHD mice will prove useful for preclinical studies of novel anti-inflammatory and immunosuppressive therapeutics, providing an important point of validation that a candidate therapeutic has activity against human cells in a complex in vivo environment. Notably, previous models of xenogenic GVHD have previously been reported [37], [38], [39], [40]. These involved transfer of human peripheral blood mononuclear cells (PBMCs) into sublethally irriadiated NS or NSG hosts, resulting in rapid (usually <2 weeks) and highly penetrant disease. We propose that PBMC models and the BLT GVH model are complimentary approaches, with the PBMC transfer model representing acute GVHD from the transfer of preformed effector T cells and the BLT GVH model more accurately representing chronic GVHD, developing over 3–4 months, arising from T cells generated *de novo* in the murine host, reflecting the contribution of multiple cell types and a multifactorial process leading to the eventual loss of host tolerance. Notably, it has been described that human T cells in similar models lacking a human thymic implant are able to home to the murine thymus to generate polyclonal TCRαβ repertoires [41]. Thus, it is likely that T cell compartments in the BLT mice reflect a mixture of cells educated in the human and murine thymus. It will be of particular interest to determine the relative pathogenic potential of each of these pools of lymphocytes.

In addition to the relevance of this model to human GVHD, it partially recapitulates pathologic features of human scleroderma, particularly with regards to the skin disease. Having a platform for preclinical validation in a humanized system is of particular importance for fibroinflammatory diseases such as scleroderma, as multiple initially promising therapeutic approaches ultimately yielded disappointing results when transitioned from preclinical models to patients [42], [43]. For future such efforts, the BLT GVHD model could serve as a final point of preclinical evaluation and optimization of a therapeutic before committing to human studies. Lastly, the BLT GVHD model may be of use in addressing clinically important questions that may be difficult to directly address in patients, such as the effect of HIV at various stages of infection on the incidence and severity of GVHD after allogenic hematopoietic stem cell transplantation [44], [45]. Thus, the BLT GVHD model can also facilitate the experimental study of questions not previously possible, such as the influence of HIV or other pathogens studied in humanized mice on GVHD.

## Supporting Information

Information S1 **Table 1,** **Figures 1** **–** **3** **.** Table 1: P values and relative risk for the influence of HLA class I alleles on GVH incidence. Relative risk and significance for various HLA class I alleles was calculated in Figure 2. Displayed are the relative risk values, 95% confidence intervals, and P values for each HLA allele. Figure 1. Further histologic characterization of GVH occurring in humanized BLT mice. (A–D)Tissues from humanized BLT mice with clinical GVH as in Figure 1 were harvested for histologic analysis. Figure 2. Cellular and molecular characterization of GVH in BLT mice. Splenocytes were isolated and stimulated as in Figure 3E, and the resulting supernatant was analyzed for levels of IL10. Figure 3: Replicate Data for Dermal Macrophage Expression Profile. Macrophages were isolated as in Figure 4, RNA extracted, and expression of the indicated genes measured by realtime PCR. Shown are independent experimental replicates of the data provided in Figure 4B.
(PDF)
