# Correction: Graft versus Host Disease in the Bone Marrow, Liver and Thymus Humanized Mouse Model

**DOI:** 10.1371/annotation/e413f2a1-5767-4c82-9e27-dd556155f124

**Published:** 2013-05-06

**Authors:** Matthew B. Greenblatt, Vladimir Vrbanac, Trevor Tivey, Kelly Tsang, Andrew M. Tager, Antonios O. Aliprantis

There was an error in the name of the second author.

The correct name is: Vladimir Vrbanac

The correct Citation is: Greenblatt MB, Vrbanac V, Tivey T, Tsang K, Tager AM, et al. (2012) Graft versus Host Disease in the Bone Marrow, Liver and Thymus Humanized Mouse Model. PLoS ONE 7(9): e44664. doi:10.1371/journal.pone.0044664 

