# Supplementary material for: Graft versus Host Disease in the Bone Marrow, Liver and Thymus Humanized Mouse Model
Source: PLoS One. 2012 Sep 5;7(9):e44664. doi: 10.1371/journal.pone.0044664 (PMC3434179; doi:10.1371/journal.pone.0044664)
Supplement: Information S1 — Table 1, Figures 1 – 3 . Table 1: P values and relative risk for the influence of HLA class I alleles on GVH incidence. Relative risk and significance for various HLA class I alleles was calculated in Figure 2. Displayed are the relative risk values, 95% confidence intervals, and P values for each HLA allele. Figure 1. Further histologic characterization of GVH occurring in humanized BLT mice. (A–D)Tissues from humanized BLT mice with clinical GVH as in Figure 1 were harvested for histologic analysis. Figure 2. Cellular and molecular characterization of GVH in BLT mice. Splenocytes were isolated and stimulated as in Figure 3E, and the resulting supernatant was analyzed for levels of IL10. Figure 3: Replicate Data for Dermal Macrophage Expression Profile. Macrophages were isolated as in Figure 4, RNA extracted, and expression of the indicated genes measured by realtime PCR. Shown are independent experimental replicates of the data provided in Figure 4B. (PDF) [file pone.0044664.s001.pdf]

Supplemental Information for

Graft Versus Host Disease in Mice Bearing Humanized Immune Systems

Matthew B. Greenblatt[1], Vladimir Vbranac [2,3] , Trevor Tivey [2,3], Kelly Tsang [1], Andrew M. Tager [2,3], Antonios O. Aliprantis[1,4]

[1] Department of Immunology and Infectious Diseases,Harvard School of Public Health, Boston, Massachusetts. [2] Center for Immunology and Inflammatory Diseases, Division of Rheumatology, Allergy and Immunology, Massachusetts General Hospital, Harvard Medical School, Charlestown, Massachusetts 02129; [3] Ragon Institute of MGH, MIT, and Harvard and Division of AIDS, Harvard Medical School, Charlestown, Massachusetts 02129; [4] Department of Medicine, Division of Rheumatology, Allergy and Immunology, Brigham and Women's Hospital and Harvard Medical School, Boston, Massachusetts, USA;

Address correspondence to: Antonios O. Aliprantis, Harvard School of Public Health, 651 Huntington Avenue, FXB 205, Boston, Massachusetts 02115, USA. Phone: 617.432.4867; Fax: 617.432.0084; E-mail: aaliprantis@partners.org.

Supplemental Table 1: P values and relative risk for the influence of HLA class I alleles on GVH incidence

Relative risk and significance for various HLA class I alleles was calculated in Figure 2. Displayed are the relative risk values, 95% confidence intervals, and P values for each HLA allele.

HLA Allele	Relative Risk	95% CI lower bound	95% CI upper bound	P Value	P Value Corrected for Multiple Comparisons
A1101	2.062	1.704	2.495	<0.0001	<.0012
A2402	2.005	1.622	2.478	<0.0001	<.0012
A3303	1.777	1.34	2.357	0.0004	0.0048
A201	1.376	1.184	1.598	<0.0001	<.0012
A206	1.297	0.8856	1.899	ns	ns
A207	1.26	0.8592	1.849	ns	ns
A301	1.257	1.043	1.517	ns	ns
A2601	1.2	0.8804	1.636	ns	ns
A6801	0.9829	0.6606	1.463	ns	ns
A101	0.9078	0.7323	1.125	ns	ns
A6802	0.5946	0.3624	0.9755	0.0287	0.3444
A3201	0.06465	0.009216	0.4535	<0.0001	<.0012

HLA Allele	Relative Risk	95% CI lower bound	95% CI upper bound	P Value	P Value Corrected for Multiple Comparisons
B1302	3.461	2.939	4.076	<0.0001	<.0012
B3502	2.74	2.187	3.432	<0.0001	<.0012
B4601	1.26	0.8592	1.849	ns	ns
B4001	1.204	0.9326	1.554	ns	ns
B3801	1.2	0.8804	1.636	ns	ns
B3501	0.9085	0.7137	1.157	ns	ns
B5101	0.8678	0.6798	1.108	ns	ns
B4403	0.8313	0.6142	1.125	ns	ns
B702	0.6919	0.5068	0.9446	ns	ns
B1510	0.5946	0.3624	0.9755	0.0387	0.4644
B801	0.4887	0.3371	0.7084	<0.0001	<.0012
B1401	0.06465	0.009216	0.4535	<0.0001	<.0012

HLA Allele	Relative Risk	95% CI lower bound	95% CI upper bound	P Value	P Value Corrected for Multiple Comparisons

C602	1.995	1.686	2.359	<0.0001	<.0009
C401	1.49	1.271	1.745	<0.0001	<.0009
C304	1.242	1.038	1.486	0.0238	0.2142
C102	1.221	0.8964	1.663	ns	ns
C1203	1.182	0.8957	1.559	ns	ns
C802	0.9427	0.626	1.419	ns	ns
C202	0.8372	0.5896	1.189	ns	ns
C702	0.7906	0.5953	1.05	ns	ns
C701	0.7383	0.5843	0.933	0.0108	0.0972

Supplemental Figure Legends

Supplemental Figure 1. Further histologic characterization of GVH occurring in humanized BLT mice.

(A-D)Tissues from humanized BLT mice with clinical GVH as in Figure 1 were harvested for histologic analysis.

Supplemental Figure 2. Cellular and molecular characterization of GVH in BLT mice.

Splenocytes were isolated and stimulated as in Figure 3E, and the resulting supernatant was analyzed for levels of IL10.

Supplemental Figure 3: Replicate Data for Dermal Macrophage Expression Profile

Macrophages were isolated as in Figure 4, RNA extracted, and expression of the indicated genes measured by realtime PCR. Shown are independent experimental replicates of the data provided in Figure 4B.

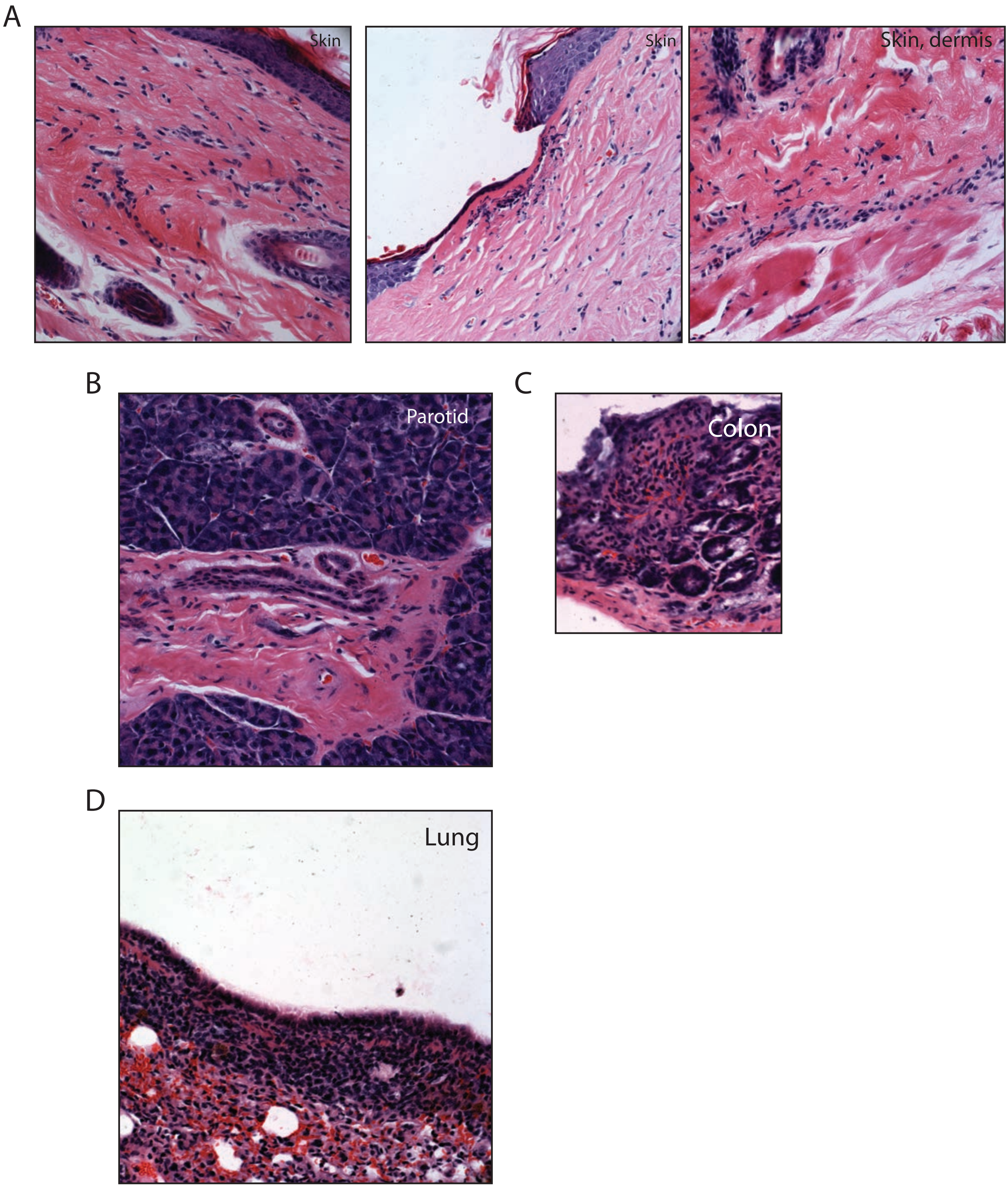

Supplementary Figure 1

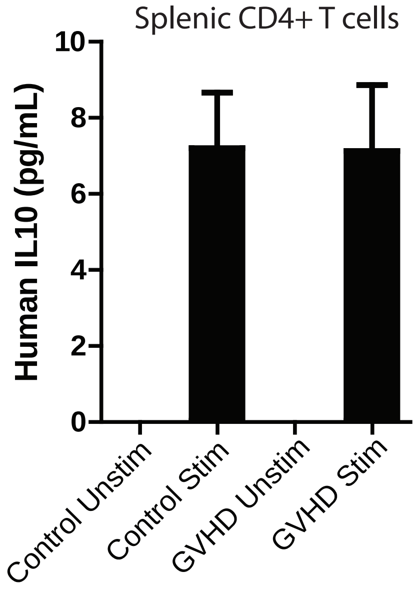

Supplementary Figure 2

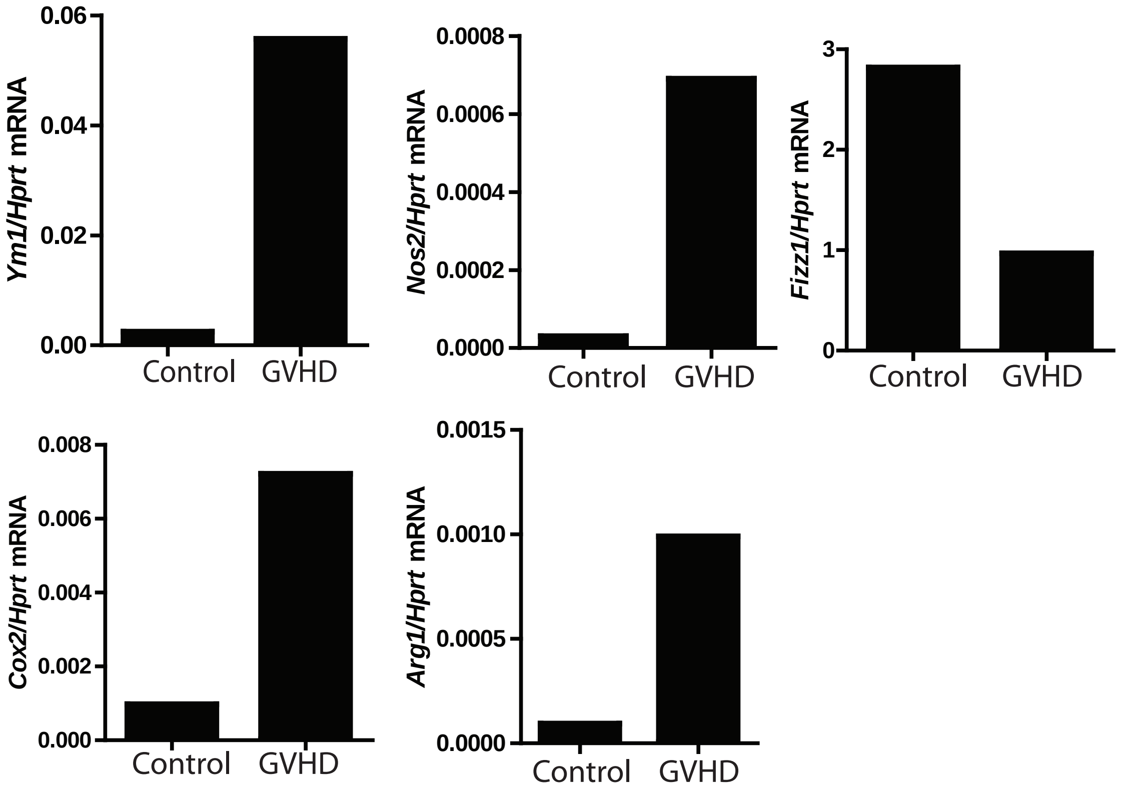

Supplementary Figure 3
